# The Week's Work

**Published:** 1910-07-02

**Authors:** 


### The Week's Work.

### THE LEAGUE OF MERCY.

His Majesty has appointed his Serene Highness Prince Alexander of Teck to be Grand President of the League of Mercy and her Royal Highness Princess Alexander of Teck to be Lady Grand President of the League. His Majesty now becomes Patron of the League and Sovereign of the Order, and has intimated to the officers of the League his intention to continue his personal interest in its welfare and to keep himself informed of the progress of its organisation.

### THE KING'S HOSPITAL FUND.

In The Hospital for June 4 we called attention to the fact that, under the Act of 1907, the presidency of this Fund would be placed in the hands of three Commissioners during the minority of the Prince of Wales. The appointment of these Commissioners is made by the Sovereign on the recommendation of the Lord Chancellor, the Prime Minister, and the Governor of the Bank of England. We are informed that the three Commissioners in whom the presidency will vest are H.S.H. the Duke of Teck, Lord Iveagh, and the Speaker of the House of Commons. Lord Iveagh has been a member of the Council of the King's Fund for some years. Successive Speakers of the House of Commons have taken a warm interest in the King's Fund and the League of Mercy, and have so materially contributed to the success of both.

### THE DREADNOUGHT SEAMEN'S HOSPITAL.

This hospital is unique amongst London voluntary hospitals, for it provides for seamen of all nations regardless of race, creed, or colour. The annual report for 1909, just issued, shows, despite every effort, that the support extended to this hospital by the public is not nearly so large as it ought to be, having regard to the fact that landsmen owe practically all their luxuries to the devotion of the sailor, and that his care and cure are matters of paramount interest to every thinking man and woman. For years past there has been an excess of expenditure over income, which in 1909 amounted in round figures to £2,340. There must surely be 3,000 people who would gladly contribute one guinea a year as an annual subscription to the sailors' hospital of the port of London, and we think the present needs of the Dreadnought should command the whole-hearted sympathy and active support of the Metropolitan and provincial Press of every shade of opinion. If a few of the great newspapers would take up the cause of the Dreadnought Hospital, £3,000 in additional annual subscriptions would be forthcoming, we are confident, without delay or difficulty. Many years ago a great provincial newspaper, the *Birmingham Daily Post*, wrote a strong article on behalf of the sailors which resulted in considerable additional revenue. The report itself is an interesting document, and has special value this year from the fact that Sir Ernest Shackleton, C.V.O., the Arctic explorer, presided at the annual meeting. His speech contains many interesting facts and a most eloquent tribute to the worth of the sailor and his claim upon landsmen in the day of sickness. We miss from the report a short history of the Dreadnought, and also of the Tropical School of Medicine, which has added lustre to the Dreadnought Hospital and done enormous good to all members of the British race who reside in the Tropics. The story of the Dreadnought and its Tropical School is one which, we think, should find a permanent place in every issue of the annual report. For the rest we would commend the management for the excellent statistical and other tables the report contains, which constitute a model that might be more generally followed in publications of this character. It is a serious task to have to raise £20,000 a year from voluntary sources for a hospital like this, and the success achieved reflects great credit upon the committee and upon Mr. Pietro Michelli, the secretary, who is one of the best known, most trusted, and influential of hospital secretaries.

### THE WORK OF HOSPITAL ALMONERS.

We have received two most interesting reports for which we tender grateful thanks to the authorities, being the Lady Almoner's Report for St. Thomas's Hospital and the Lady Almoners' Report of the London Hospital. It is not possible in this column to do justice to these two able documents, which are full of facts, and we propose to deal with them at length in a separate article. We hope these reports will be sent out to the general Press all over the country after the rising of Parliament, when the editors will have less demand upon their space, for then, we believe, they must attract wide interest and result in additional support and sympathy for the work of the two hospitals immediately concerned. These reports abundantly justify the policy of King Edward's Hospital Fund in insisting that every hospital shall employ at least one hospital almoner who shall devote the whole of his or her time to the work. Our voluntary hospitals render an immense service to the sick poor by affording the best medical and surgical aid and hospital maintenance. The debt which the public owe to the voluntary hospitals for work of this kind is being immeasurably increased by the work of hospital almoners like those referred to, for their reports contain abundant evidence that the authorities of each of these hospitals conduct their establishments on the fundamental idea that the great object of a voluntary hospital should be to secure the lasting good of the patient. When, as in the case of the London Hospital, the almoners are fully alive to the fact that aid, other than hospital treatment, should only be given when it is likely to prove of some permanent value in building up the moral character of the patient, the innovation which an almoner's appointment represents adds immeasurably to the value of the work done for the poor by every well-administered voluntary hospital.

### A BAD PRECEDENT.

We have always supported the Hospital Saturday Fund and its journal, and we regret to see that in the last issue of the latter, dated June 30, 1910, in an article entitled "King Edward VII.," material portions of the article which appeared in our Hospital Sunday number of June 12 on the same subject have been appropriated without acknowledgment of any kind. Considering that the "lifted" portions contain facts never before published, and that the article in question suppresses other material facts which are a matter of history, we hope that no repetition of the practice here referred to may be made for the future. We would especially draw the attention of Sir Savile Crossley, the Chairman of the Hospital Saturday Fund, to this matter, because he is seised of the facts, and would, we believe, heartily discountenance such practices.

### CHICHESTER INFIRMARY.

In our issue of October 9 we published a description of this Infirmary, in which the old buildings were commended as possibly the best specimen extant of the old H plan of construction. It was pointed out that a sum of £500 might be usefully expended in new fittings and the latest appliances in lieu of several which, though possibly sufficient, are not sightly or modern, and so should be removed. It was further stated that a few structural defects can be readily remedied. Bearing these facts in view it is not a little remarkable that the Bishop of Chichester should have recently issued an appeal in the county papers for £20,000, which, from inquiries we have made, it would appear it is proposed to expend upon the erection of a new operation-room and a reconstruction of the sanitary blocks. We have no hesitation in saying that to expend anything like £20,000 upon such a simple reconstruction would be a waste of public money, and we hope the committee of the Chichester Infirmary will reconsider the whole plan so as to reduce the proposed expenditure to reasonable proportions. The Chichester Infirmary has no great demand upon its beds. The residents in Sussex have recently come forward, as we suggested, and paid off a debt of some £2,000, and we venture to say it is neither wise, prudent, nor justifiable to ask for so large a sum as £20,000 for reconstruction purposes in connection with this excellent hospital at the present time. The architect entrusted with the work is described as "the well-known expert in hospital architecture," and we should be glad to have information as to what new hospitals he has planned, and to have a list of the hospital work which has been carried out from his designs.

### MIDDLESEX HOSPITAL.

In another column we publish a letter of appeal on behalf of this hospital from Prince Francis of Teck. Prince Francis, who has undertaken the duties of Chairman of this hospital, is showing commendable energy and zeal in raising the sum of £20,000 which is "immediately required to save the hospital from a dangerous position." Founded in 1745 and incorporated in 1836, Middlesex Hospital has within the last thirty years been so largely reconstructed and added to that the present buildings represent many features of a modern hospital. It has a large cancer-department, which has been the means of alleviating much human misery by receiving into its wards incurable cases, where they have received the kindest and fullest attention, and have so been enabled to end their days under conditions which have minimised their sufferings to the fullest possible extent. We hope that the appeal of H.S.H. will be readily and generously responded to, and that the £20,000 may speedily be forthcoming. Apart altogether from the merits of this particular appeal, the action of Prince Francis of Teck in following the example of King Edward by giving so large a measure of personal service to the cause of the sick cannot fail to lead many others to go and do likewise. The voluntary hospitals never wanted the help which men of influence can render them in the administration and management of their financial affairs more than they do at the present time. Who can over-estimate the value of such services as have been rendered to individual hospitals by lovers of hospital work like Mr. Cosmo Bonsor; Mr. Ralph Brocklebank, of Liverpool; Sir William Cobbett, of Manchester; Mr. Wade Deacon, of Liverpool; Mr. Alfred Herbert, of Coventry; the Hon. Sydney Holland; Sir Riley Lord, of Newcastle; and Mr. Charles Lupton, of Leeds; to name only a few of the many prominent workers who practise the doctrine of personal service in the days of health in the cause of the sick?

### THE DRUG MARKET.

Business continues very dull, with few noteworthy alterations in price. Santonin has been advanced 1s. 6d. per lb., and a further advance is expected to be made in a few days. Buchu leaves, of the best quality, are practically unobtainable, and an exceedingly high price has been paid for a small quantity. The position of bromides is as yet unaltered. A slight reduction in the price of morphine should not be entirely unexpected. Jalap continues very dear, with a tendency to advance. Cod-liver oil has again declined in value to a slight extent, and the demand continues extremely quiet.

